# Highly Cited Articles in Evolutionary Psychiatry: Assessment With a Quality and Error Rating Scale

**DOI:** 10.31083/AP39276

**Published:** 2025-12-24

**Authors:** Chad Beyer, Chanel Robinson, Dan J. Stein

**Affiliations:** ^1^Department of Psychiatry, University of Cape Town, 7701 Cape Town, South Africa; ^2^Western Cape Department of Health, 7701 Cape Town, South Africa; ^3^SAMRC Unit on Risk & Resilience in Mental Disorders, Department of Psychiatry & Neuroscience Institute, University of Cape Town, 7701 Cape Town, South Africa

**Keywords:** evolutionary psychiatry, systematic review, citation analysis, article quality, common errors, rating scale

## Abstract

**Background:**

Evolutionary psychiatry is a growing field that emphasizes the value of evolutionary explanations for traits that make individuals vulnerable to mental disorders. Some articles that apply evolutionary theory in psychiatry make errors, such as viewing a disease as an adaptation. We assessed article quality and error quantity in the most cited articles on evolutionary psychiatry and examined the relationship of these measures to citation number.

**Methods:**

PubMed, Web of Science, and Google Scholar were searched in 2023 and again in 2025 using specific terms related to “evolution” and “psychiatry”, to find the most highly cited articles in the field. Based on the work of Nesse, we developed a measure for assessing overall article quality and error quantity in evolutionary psychiatry articles. We applied the measure to the 20 most highly cited articles, and calculated the correlations of article quality and error quantity with number of citations.

**Results:**

Twenty highly cited articles, with a mean citation count of 413.30 and publication year from 1964 to 2011, were rated. While the most highly cited articles had good quality on average, they also made important errors. There was no significant correlation of article quality or error quantity with citation count.

**Conclusion:**

Highly cited articles in evolutionary psychiatry demonstrated strengths but also weaknesses. The lack of a relationship of article quality and error quantity to citation rates suggests that other factors influence such citations. Future research should focus on achieving consensus on how best to assess the quality of evolutionary psychiatry articles and what errors should be avoided.

## Main Points

1. The most highly cited articles in evolutionary psychiatry demonstrated overall good quality, particularly in defining research questions with precision.

2. Important conceptual errors were nevertheless common, with half of the top-cited articles failing to consider alternative hypotheses or presenting unbalanced evidence.

3. No significant relationship was found between article quality, error quantity, and citation count, suggesting citation frequency is driven by factors other than scientific rigor.

4. The rating system derived from Nesse’s quality and error criteria showed reliable application, supporting its potential utility as a standardised evaluative tool for the field.

5. Findings highlight a need for consensus on quality standards in evolutionary psychiatry, as well as improved methodological rigour in specifying and evaluating evolutionary hypotheses.

## 1. Introduction

The seminal text, “Why We Get Sick” argued that evolutionary theory is useful in understanding traits that leave members of a species vulnerable to disease [1]. Evolutionary medicine and evolutionary psychiatry subsequently emerged as new disciplines, with specialized conferences, textbooks, and journals [2]. A recent review of evolutionary psychiatry updated the range of possible explanations for vulnerability to mental disorders including mismatch of evolutionary history with current environments and evolutionary tradeoffs that have both benefits and costs [3]. Such explanations complement proximal biological explanations that describe mechanisms underlying disease with more distal evolutionary accounts of the origins of vulnerability to failure [3].

Evolutionary medicine and psychiatry have grappled with the conceptual questions of what makes for a high quality publication in the field, and of what conceptual errors continue to be made by contributors [4]. It has been suggested that good articles in the field define their questions with precision and consider multiple possible explanations of observed phenomena [5]. In contrast, errors include viewing disorders as adaptations, and providing explanations based on what is good for the species [4]. The difficulty in delineating normality from psychopathology is a particular problem in psychiatry and evolutionary psychiatry, where the drawing of a line between normal and excessive activation of an adaptive defence often becomes blurry [6]. This article is an effort towards providing some degree of quality standardization for the field.

We developed a measure of the overall quality and error number in evolutionary psychiatry articles, based on Nesse’s work on how to test evolutionary hypotheses, and on errors in the field [4]. Nesse’s work on quality standards is in turn based on extensive engagement with and review of the field. We applied these measures to the most highly cited articles in the field. We assessed article quality and error quantity in the most cited articles on evolutionary psychiatry and examined the relationship of these measures to citation frequency.

## 2. Methods

### 2.1 Selection of Studies

Two reviewers (CB and CR) searched Pubmed (https://pubmed.ncbi.nlm.nih.gov/), Web of Science (https://www.webofscience.com) and Google Scholar (https://scholar.google.com/) on 8 September 2023 and repeated the search on 7 January 2025 for relevant articles using search terms curated for each database, covering “evolution” and “psychiatry”. MeSH terms (https://www.ncbi.nlm.nih.gov/mesh/) were primarily used, however, as most databases do not allow ordering by citation count, this method was augmented by searching reference lists of review articles.

Two authors (CB and CR) reviewed the titles and abstracts of articles obtained by this search strategy. Included in this review were all articles that made evolutionary claims about particular mental disorders, excluding review articles, unless they made specific claims, and those focused on non-human research. The 20 most highly cited studies were included in this study.

### 2.2 Rating of Studies

Articles were assessed for quality based on the article “How to test an evolutionary hypothesis about disease” [4]. This article lists 4 main objectives, namely: F1—Define the object of explanation with great specificity; F2—Specify all possible alternative hypotheses for why the trait is apparently suboptimal; F3—Make explicit predictions from each hypothesis; F4—Use all available evidence to test the predictions from all alternative hypotheses to arrive at a judgment about the contributions of different factors.

To generate a total quality score (TQS), each article was rated on each of the four objectives using a Likert scale ranging from 1 to 5: to what extent do you believe this paper adequately addresses the following, 1—Strongly disagree; 2—Disagree; 3—Neither agree nor disagree (uncertain); 4—Agree; 5—Strongly agree. The four scores were summed to give a TQS of 4 to 20. This score was then divided by 4 and anchored as follows 1–1.8 = very poor; 1.8–2.6 = poor; 2.6–3.4 = average; 3.4–4.2 = good; 4.2–5.0 = very good.

Articles were then assessed for errors based on the article “Some common mistakes in testing evolutionary hypotheses about disease” [4]. These errors are paraphrased here: FN1—Attempting to explain a disease as if it is an adaptation; FN2—Proposing an explanation based on what is good for the species; FN3—Proposing adaptive functions for rare genetic conditions; FN4—Confusing proximate and evolutionary explanations; FN5—Thinking that evidence for learning influencing a trait indicates that no evolutionary explanation is needed; FN6—Thinking that evidence for environmental or cultural differences in a trait is evidence against evolutionary influences; FN7—Confusing genetic explanations, especially behavioural genetic explanations, with evolutionary explanations; FN8—Failing to consider all of the alternative hypotheses; FN9—Assuming that evidence for one hypothesis is evidence against another; FN10—Presenting all the evidence in favour of a pet hypothesis and all the evidence against other hypotheses, instead of offering a balanced consideration of all evidence for and against all hypotheses.

We assessed each article for evidence of whether each of these errors was made. As points 1–4 above were viewed as more fundamental errors than points 5–10, errors from points 1–4 were each given an error score of 1, while errors from points 5–10 were each given an error score of 0.5. Thus the total error score (TES) could range from 2 to 7.

Finally, each article was assigned an Overall Impression (or global rating) that ranged from 1 (Poor) to 5 (Excellent) (Table 1).

**Table 1.** **Final global impression (FGI) scoring**.

1 Poor	Makes an elementary mistake about evolutionary theory (attempts to explain a disease, proposes an explanation based on what is good for the species, proposes adaptive functions for rare genetic conditions, confuses proximate and evolutionary explanations).
2 Problematic	Makes no elementary mistakes about evolutionary theory, but does not represent the best of evolutionary theory (focused on proximal explanations rather than evolutionary ones).
3 Middling	Is consistent with good evolutionary theory, but defines the object of explanation with limited specificity, with limited discussion of alternate hypotheses or explicit predictions, or without clearly demonstrating clinical implications.
4 Good	Defines the object of explanation with some specificity, and has some discussion of alternative hypothesis or explicit predictions, and has clear discussion of the clinical implications.
5 Excellent	Defines the object of explanation with great specificity (a trait shaped by natural selection, that makes an organism vulnerable to disease), specifies alternate hypotheses about why the trait is suboptimal, make explicit predictions from the hypotheses, and if true promises to change clinical practice.

Final global impression composing aspects of both the positive and negative scores (range from 1 (Poor) to 5 (Excellent)).

All articles were scored independently by 2 reviewers (CB, CR). Differences were resolved by discussion or by bringing in a third reviewer (DJS).

### 2.3 Approach

Article quality (as measured by TQS), error quantity (as measured by TES), and Overall Impression scores were subjected to Cohen’s Kappa to assess inter-rater reliability (IRR), prior to resolution by discussion or the third reviewer (DJS). These results were interpreted as follows: values ≤0 as indicating no agreement and 0.01–0.20 as none to slight, 0.21–0.40 as fair, 0.41–0.60 as moderate, 0.61–0.80 as substantial, and 0.81–1.00 as almost perfect agreement.

Article quality (as measured by TQS), error quantity (as measured by TES), and Overall Impression scores for each article were correlated with citation count, with significance assessed using a two-tailed *t*-test. Spearman correlation was used for data that were non-normally distributed, of ordinal scale (i.e., publication year), or when the relationship between variables was non-linear. Statistical analysis was performed using IBM SPSS Statistics (version 26.0, IBM, Armonk, NY, USA) and differences were considered significant where *p* < 0.05.

## 3. Results

### 3.1 Included Studies

We analyzed the 20 most highly cited articles in evolutionary psychiatry [7, 8, 9, 10, 11, 12, 13, 14, 15, 16, 17, 18, 19, 20, 21, 22, 23, 24, 25, 26]. The lowest cited article had 130 citations, while the highest cited article had 1000 citations, with a mean of 413.30, median of 362, standard deviation of 250.52, and range of 870. The earliest published article was from 1964, with the most recent article from 2011, with a mean publication year of 2000 and a standard deviation of 10.01. Table 2 (Ref. [7, 8, 9, 10, 11, 12, 13, 14, 15, 16, 17, 18, 19, 20, 21, 22, 23, 24, 25, 26]) shows the included articles, with study ID, Scopus citation count and Field-Weighted Citation Impact (FWCI) where available.

**Table 2.** **Included articles**.

Article ID	Citations	Pub. Year
Allen and Badcock [7]	340	2003
Andrews and Thomson [8]	382	2009
Bateson *et al*. [9]	146	2011
Brüne [10]	850	2005
Crespi and Badcock [11]	435	2008
Crow [12]	307	2000
Gilbert and Allan [13]	655	1998
Hagen [14]	183	1999
Huxley *et al*. [15]	188	1964
Jonason *et al*. [16]	626	2009
Klein [17]	1000	1993
Marks and Nesse [18]	448	1994
Mealey [22]	676	1995
Nesse [19]	130	1998
Nesse [20]	625	2000
Nesse [21]	309	2005
Nettle and Clegg [23]	204	2006
Price *et al*. [24]	405	1994
Sloman *et al*. [25]	170	2003
Watson and Andrews [26]	187	2002

Table 2 shows the included articles, with study ID and citation number as per Scopus (Current as of 07/01/2025).

### 3.2 Study Ratings

Inter-rater reliability was assessed using Cohen’s Kappa, with selected values reported for key scoring domains. Across all domains, there was an overall agreement rate of 68.5%.

**Fig. 1.**
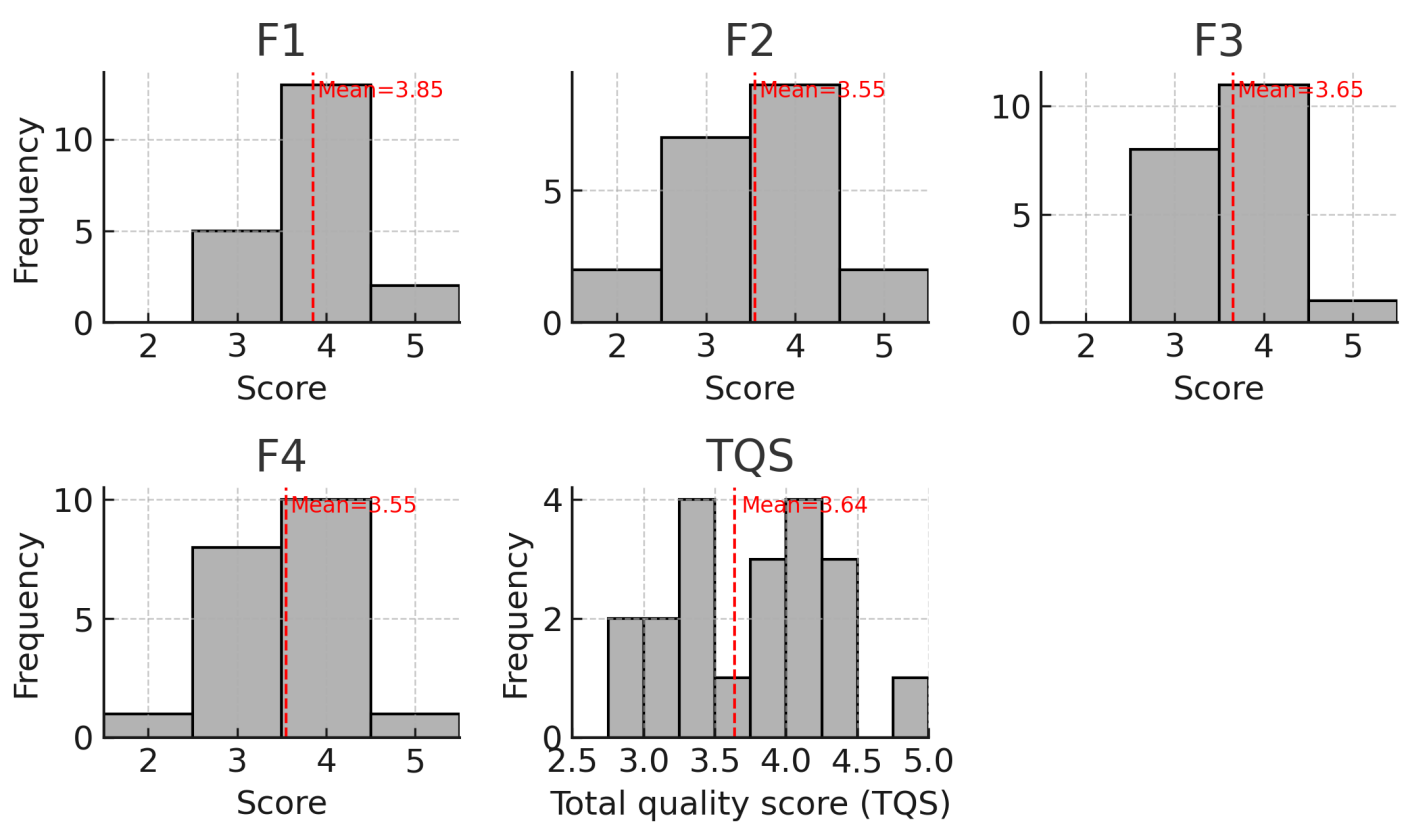
**Distributions of domain scores (F1–F4) and total quality score (TQS)**. Fig. 1 contains multiple histograms which demonstrate the distribution of the various domain scores as well as the total quality score, with the mean of each presented with a dotted line.

Fig. 1 shows the distributions of positive scores (F1–F4) and TQS. Histograms display the frequency of reviewer scores assigned to each quality domain (F1–F4) and the TQS across the 20 included articles. Scores for F1–F4 were recorded on a 1–5 scale; TQS is the mean of the four domain scores. The overall distribution of TQS shows clustering toward both moderate (≈3) and higher (≈4+) values. The overall TQS showed a mean of 3.6, standard deviation of 0.6. This falls on the low end of the good score of 3.4–4.2. Notably, the highest scoring section was F1 (M = 3.85, SD = 0.6). There were varying degrees of IRR, with F4 showing the highest level (k = 0.4).

Table 3 shows the results of the error scoring, with number of times the error was made and the percentage. Notably, FN8 and FN10 were found to be the most common errors, each made by ten of the articles assessed (50%). FN5 and FN6 were not found to be made in the assessed articles. The item with the highest IRR was FN1 (k = 0.857).

**Table 3.** **Prevalence of conceptual errors across articles**.

Error (brief description)	Articles with error (n)	% of articles (N = 20)
FN8 (Ignore alternative hypotheses)	10	50%
FN10 (Unbalanced evidence for pet hypothesis)	10	50%
FN1 (Explain disease as adaptation)	4	20%
FN9 (Evidence for A = evidence against B)	4	20%
FN3 (Adaptive function for rare genetic condition)	3	15%
FN4 (Mix proximate vs evolutionary)	2	10%
FN7 (Genetic ≠ evolutionary explanation)	2	10%
FN2 (Species-level good reasoning)	1	5%
FN5 (Learning evidence = no evolution)	0	0%
FN6 (Env/cultural variation = anti-evolution)	0	0%

Percentages are based on N = 20 articles. Errors are listed in descending order of prevalence. Brief descriptions in parentheses.

The mean Overall Impression score was 3.0, with a standard deviation of 1.5. There was moderate agreement in terms of IRR (k = 0.6).

### 3.3 Results

**Fig. 2.**
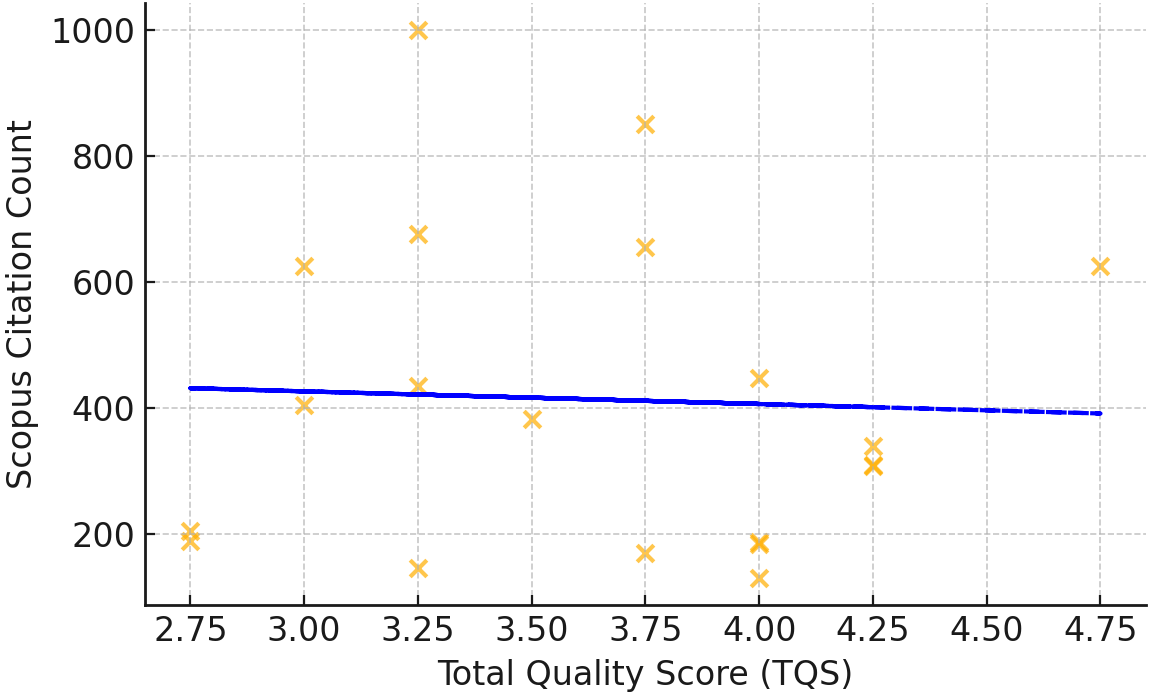
**Quality score vs scopus citation count**. Fig. 2 is a scatterplot which demonstrates that there was no correlation found between total quality score and scopus citation count.

**Fig. 3.**
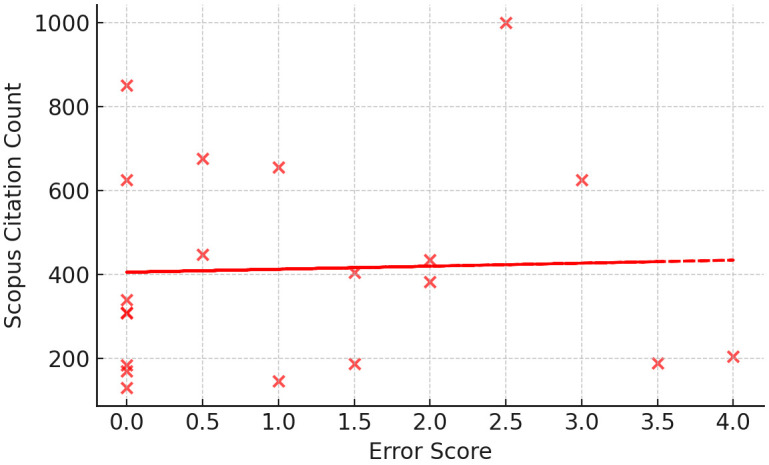
**Error score vs scopus citation count**. Fig. 3 is a scatterplot which demonstrates that there was no correlation found between error score and scopus citation count.

There was no significant correlation between TQS and either citation count (ρ = –0.123, *p* = 0.605, n = 20) or field weighted citation impact (FWCI) (ρ = –0.204, *p* = 0.465, n = 15). The error quantity was not significantly related to citation count (ρ = 0.332, *p* = 0.152, n = 20) or FWCI (ρ = 0.254, *p* = 0.360, n = 15). The Overall Impression score was likewise unrelated to citation count (ρ = –0.168, *p* = 0.478, n = 20) or FWCI (ρ = –0.137, *p* = 0.627, n = 15). Fig. 2 is a scatterplot which depicts the relationship TQS and citation count, while Fig. 3 is a scatterplot which depicts the relationship between TES and citation count.

The reviewer rating scales were strongly inter‑correlated: TQS was associated with lower TES (ρ = –0.700, *p* = 0.001, n = 20) and with better Overall Impression (ρ = 0.770, *p* < 0.001, n = 20). Higher TQS was associated with poorer Overall Impression (ρ = –0.726, *p* < 0.001, n = 20).

Publication year did not correlate with citation number (ρ = –0.187, *p* = 0.431, n = 20) or FWCI (ρ = 0.232, *p* = 0.405, n = 15).

As expected, citation number and FWCI were strongly positively related (ρ= 0.800, *p* < 0.001, n = 15).

## 4. Discussion

The main findings of this research were (1) the most highly cited articles have good quality on average, (2) the most highly cited articles make important errors, (3) there was no significant relationship between article quality or error quantity and citation number.

Overall, the top 20 mostly highly cited articles achieved a mean on the low end of the “good” rating for each of the 4 positive scoring metrics (3.6–3.9; with good considered 3.4–4.2). For example, the TQS had a rating of 3.64 (SD 0.6). The included articles scored a mean of 3.85 on F1 (Define the object of explanation with great specificity), indicating that this area represents a strength of the included articles. Conversely, F3 (Make explicit predictions from each possible hypothesis) had a mean of 3.60, while F2 (Specify all possible alternative hypotheses for why the trait is apparently suboptimal) and F4 (Use all available evidence to test the predictions from all alternative hypotheses) had the lowest mean score of included articles (3.55), indicating that these are areas that future articles can improve upon.

Of the errors made, the most common were failing to consider all of the alternative hypotheses and presenting all the evidence in favour of a pet hypothesis and all the evidence against other hypotheses, instead of offering a balanced consideration of all evidence for and against all hypotheses. 50% of the assessed articles made one or both of these errors.

None of the top 20 articles most highly cited articles made the errors of FN5 (Thinking that evidence for learning influencing a trait indicates that no evolutionary explanation is needed) or FN6 (Thinking that evidence for environmental or cultural differences in a trait is evidence against evolutionary influences). These errors may, however, be more prevalent in less cited evolutionary psychiatry articles.

Article quality and error quantity were not significantly associated with citation number. This is consistent with previous work in many fields suggesting that quality and methodological rigor are not necessarily correlated with citation number [27, 28]. In neuroscience, orthopaedics and plastic surgery citation numbers are influenced by factors including age of the article, study design, level of evidence, conflict of interest disclosures, and number of authors, but our study was not designed to investigate such influence [27, 29, 30].

This article indicates that the quality standards developed by Nesse can be assessed reliably, and suggests that their routine application could assist the field. Nesse’s quality standards have face validity insofar as they align with general scientific procedures, and our assessment measure now adds procedural validity insofar as it potentially allows different articles to be rated in a standard way. We cannot provide external validity of this measure against a pre-existing measure as this is the first such measure in the field, and indeed the question of how best to define the optimal “gold standard” for assessing the quality of work in evolutionary psychiatry is perhaps an “essentially contested” question that deserves ongoing attention.

Several additional limitations deserve emphasis. First, we assessed only the top 20 highly cited articles, which limits generalizability to the broader field of evolutionary psychiatry; it is possible that less highly cited articles would receive quite different quality and error scores. Second, we relied solely on Scopus for citation numbers; different databases provide different citation numbers, and may have yielded a different selection of articles. Third, some of the scoring items, particularly FN 1–4 and Overall Impression, involve a degree of subjective judgment; more objective anchoring of scores may be possible in the future. Fourth, we used one particular weighting system for summing error quantity; other weightings could be more useful and may be worth investigating. Fifth, the raters are both physicians rather than experts in evolutionary theory or medicine, and the high kappas obtained may not be generalizable to other raters. Sixth, we relied heavily on the work of Nesse; additional or different criteria may also be useful.

## 5. Conclusion

Our results suggest that the most highly cited articles in evolutionary psychiatry have both strengths and weaknesses. Strengths include defining the research question with precision and predicting the answers based on a specific hypothesis. Weaknesses include failing to consider and specify alternate hypotheses, and presenting evidence in favour of a pet hypothesis while not using all available evidence. The lack of an association of article quality and error quantity with citation number suggests that other factors influence such citations. While the field has significant promise, further work is needed to achieve consensus on how best to assess the quality of evolutionary psychiatry articles and on what errors should be avoided.

## Availability of Data and Materials

Data is available from the authors.
